# AmcA—a putative mitochondrial ornithine transporter supporting fungal siderophore biosynthesis

**DOI:** 10.3389/fmicb.2015.00252

**Published:** 2015-04-07

**Authors:** Lukas Schafferer, Nicola Beckmann, Ulrike Binder, Gerald Brosch, Hubertus Haas

**Affiliations:** ^1^Division of Molecular Biology/Biocenter, Medical University InnsbruckInnsbruck, Austria; ^2^Division of Hygiene and Medical Microbiology, Medical University InnsbruckInnsbruck, Austria

**Keywords:** *Aspergillus fumigatus*, AmcA, iron, siderophores, ornithine, mitochondria, virulence

## Abstract

Iron is an essential nutrient required for a wide range of cellular processes. The opportunistic fungal pathogen *Aspergillus fumigatus* employs low-molecular mass iron-specific chelators, termed siderophores, for uptake, storage and intracellular iron distribution, which play a crucial role in the pathogenicity of this fungus. Siderophore biosynthesis (SB) depends on coordination with the supply of its precursor ornithine, produced mitochondrially from glutamate or cytosolically via hydrolysis of arginine. In this study, we demonstrate a role of the putative mitochondrial transporter AmcA (AFUA_8G02760) in SB of *A. fumigatus.* Consistent with a role in cellular ornithine handling, AmcA-deficiency resulted in decreased cellular ornithine and arginine contents as well as decreased siderophore production on medium containing glutamine as the sole nitrogen source. In support, arginine and ornithine as nitrogen sources did not impact SB due to cytosolic ornithine availability. As revealed by Northern blot analysis, transcript levels of siderophore biosynthetic genes were unresponsive to the cellular ornithine level. In contrast to siderophore production, AmcA deficiency did only mildly decrease the cellular polyamine content, demonstrating cellular prioritization of ornithine use. Nevertheless, AmcA-deficiency increased the susceptibility of *A. fumigatus* to the polyamine biosynthesis inhibitor eflornithine, most likely due to the decreased ornithine pool. AmcA-deficiency decreased the growth rate particularly on ornithine as the sole nitrogen source during iron starvation and sufficiency, indicating an additional role in the metabolism and fitness of *A. fumigatus*, possibly in mitochondrial ornithine import. In the *Galleria mellonella* infection model, AmcA-deficiency did not affect virulence of *A. fumigatus*, most likely due to the residual siderophore production and arginine availability in this host niche.

## Introduction

Iron is an essential nutrient for virtually every organism known to mankind. Its ability to adopt one of two ionic forms, reduced ferrous (Fe^2+^) or oxidized ferric (Fe^3+^) iron, makes it the major redox metal in cells. Although abundant in the earth's crust, its bioavailability is very low. The oxidized form, or more accurately oxyhydroxide colloid particles, is found in aerobic environments and shows a solubility below 10^−9^ M at neutral pH, which is insufficient to sustain vital processes (Ratledge and Dover, 2000). Apart from its crucial role in metabolism, including respiration, oxidative stress detoxification as well as synthesis of amino acids, lipids and desoxyribonucleic acid, iron is able to generate toxic reactive species if accumulated excessively (Halliwell and Gutteridge, 1984). As a result, organisms have developed fine-tuned regulatory mechanisms regarding uptake, storage and consumption of iron.

The ascomycete *Aspergillus fumigatus* exhibits a typical saprophytic lifestyle and is usually found in soil and decaying matter. Nevertheless, it has become the most common airborne, pathogenic fungus causing life-threatening disease in immuno-compromised patients. While it lacks specific uptake systems for host iron, it employs two high-affinity iron uptake systems; reductive iron assimilation (RIA) and siderophore-mediated iron uptake. Previous studies have demonstrated a crucial role of siderophores in virulence of *A. fumigatus*, as the elimination of both extra- and intracellular siderophores resulted in absolute avirulence in a mouse model of pulmonary aspergillosis (Schrettl et al., 2004).

Siderophores are small, high-affinity iron chelating compounds found in bacteria, fungi and plants. These molecules form a complex with Fe^3+^ to overcome the aforementioned bioavailability problem or to extract iron from host proteins. In the case of *A. fumigatus*, four different hydroxamate siderophores are produced: extracellular fusarinine C (FsC) and triacetylfusarinine C (TAFC) to mobilize extracellular iron, as well as intracellular ferricrocin (FC) for hyphal storage and distribution of iron, and hydroxyferricrocin (HFC) for conidial iron storage (Schrettl et al., 2007; Wallner et al., 2009).

FsC is the prototype of fusarinines and is made up of three *N*^*5*^-*cis*-anhydromevalonyl-*N*^5^-hydroxyornithine residues, which are linked cyclically by ester bonds (Haas et al., 2008). TAFC is derived from FsC by its tri-*N*^2^-acetylation. FC is a cyclic hexapeptide of three *N*^*5*^-acyl-*N*^*5*^-hydroxyornthines and three amino acids (glycine, serine, or alanine), and HFC is the hydroxylated FC (Haas et al., 2008). Biosynthesis of all four siderophore-types starts with the *N*^*5*^-hydroxylation of ornithine, which is catalyzed by the ornithine-*N*^*5*^-monooxygenase SidA (Schrettl et al., 2004). This initial step is followed by *N*^*5*^-acylation of *N*^*5*^-hydroxyornithine to form the hydroxamate group (Haas et al., 2008). At this point, the biosynthesis pathway splits into two different pathways, depending on the choice of the acyl group (Schrettl et al., 2007). Biosynthesis of FC and HFC requires the addition of acetyl, mediated by the transacylase SidL and an uncharacterized enzyme (Blatzer et al., 2011). For formation of fusarinines, the addition of anhydromevalonyl catalyzed by the transacylase SidF is needed (Schrettl et al., 2007). In the next step, the hydroxamate and additional amino acid residues are covalently linked via ester or peptide bonds, which are achieved by nonribosomal peptide synthetases, SidC and SidD, resulting in FC and FsC (Schrettl et al., 2007).

The major precursor for all *A. fumigatus* siderophores is the non-proteinogenic amino acid ornithine, which is additionally involved in arginine metabolism, the urea cycle and polyamine biosynthesis. Ornithine can be produced in mitochondria or the cytosol (Schrettl et al., 2010; Beckmann et al., 2013). Within mitochondria, ornithine is synthesized from glutamate involving six enzymes. Subsequently, it is transported into the cytoplasm or converted to citrulline by the ornithine transcarbamoyl transferase ArgB (Jadoun et al., 2004), which is shuttled into the cytoplasm. In the cytoplasm, citrulline is converted via three enzymatic steps to arginine, which can be hydrolyzed to ornithine. A scheme of the enzymatic links between arginine/ornithine, polyamine and siderophore metabolism is depicted in **Figure 7** (Schrettl et al., 2010; Haas, 2012; Beckmann et al., 2013). In order to characterize the ornithine supply for siderophore biosynthesis (SB) in *A. fumigatus*, we aimed to identify the mitochondrial ornithine exporter.

*Saccharomyces cerevisiae* Arg11, a member of the mitochondrial carrier protein family, was suggested to play a role in arginine biosynthesis, either by importing glutamate into the mitochondrion or by exporting ornithine from the organelle to the cytosol (Crabeel et al., 1996). Further studies indicated that its main role is the transport of ornithine across the membrane into the cytosol (Palmieri et al., 1997). The *Neurospora crassa* ortholog Arg13 was similarly found to be involved in arginine metabolism, transporting ornithine from the cytosol into mitochondria or the other way around (Liu and Dunlap, 1996). Deficiency in the human ortholog ORNT1 is associated with hyperornithinaemia-hyperammonaemia-homocitrullinuria syndrome (HHH syndrome), an autosomal recessive disease with persistent hyperornithinaemia and episodic hyperammonaemia indicating that ORNT1 is crucial for transport of ornithine from the cytosol into the mitochondria, enabling proper urea cycle function as well as degradation of ornithine (Camacho et al., 1999).

The *A. fumigatus* homolog of *S. cerevisiae* Arg11 and *N. crassa* Arg13 was found to be transcriptionally upregulated under iron starvation (with glutamine as nitrogen source) in *Aspergillus nidulans* as well as *A. fumigatus* and subsequently termed AmcA (Oberegger et al., 2001; Schrettl et al., 2008, 2010). These studies indicated a role of the two major iron regulatory transcription factors in control of *amcA* expression: iron-repression mediated by the GATA-transcription factor SreA and activation during iron starvation by the bZip transcription factor HapX are involved in the transcriptional control of this mitochondrial ornithine transporter in *A. fumigatus*.

We previously demonstrated that the supply with the precursor ornithine plays an important role in siderophore production (Schrettl et al., 2010; Beckmann et al., 2013). Furthermore, studies employing arginine auxotrophic mutants indicated that SB is mainly fueled by mitochondrial rather than cytosolic ornithine production, at least with glutamine as nitrogen source (Beckmann et al., 2013). Here, we characterized the role of AmcA in general viability of *A. fumigatus* as well as its function in siderophore production on different nitrogen sources.

## Materials and methods

### Fungal strains and growth conditions

*A. fumigatus* strains were grown at 37°C in *Aspergillus* minimal medium containing 1% glucose as carbon source and 20 mM glutamine as nitrogen source (Pontecorvo et al., 1953). The use of other nitrogen sources is indicated in the text. Iron-replete media contained 30 μM FeSO_4_. For iron depleted conditions, iron was omitted. Bathophenanthroline disulfonate (BPS) was added to a concentration of 200 μM. For growth assays, 10^4^ and 10^8^ conidia were used for point-inoculation on plates or inoculation of 100 ml liquid media, respectively. Fungal strains used in this study are listed in Table S1.

### Analysis of siderophores, free amino acids and polyamines

Analysis of the free amino acid content was obtained by ethanol extraction and subsequent reversed-phase HPLC as described previously (Berger et al., 2008). Intracellular siderophores were analyzed from cell extracts as described previously (Schrettl et al., 2010). Extracellular siderophores were analyzed via reversed-phase HPLC. Chromatographic analyses were performed on a Dionex UltiMate 3000 HPLC system (Thermo Scientific, Waltham, Massachusetts, USA) with a diode array detector and a Nucleosil 100-5 C_18_ reversed phase column (250 mm × 4.6 mm I.D.; particle size 5.0 μm; Macherey-Nagel, Düren, Germany). A gradient elution was used at a constant flow rate of 0.5 ml/min with a mobile phase of water and 0.1% TFA (solvent A) and 85% acetonitril and 0.1% TFA (solvent B) starting at 6% B. For elution of siderophores, the following conditions were applied: 6–15% B during 10 min, 16–60% B during 15 min, 60% B for 5 min, 100% B for 5 min, return to 6% B for re-equilibration. For quantification of polyamines, 50 mg freeze-dried mycelia were homogenized and incubated with 6% perchloric acid for 3 h. Polyamine derivatization was carried out a ccording to Wongyai et al. (1989) with slight modifications (Beckmann et al., 2013).

### DNA, RNA isolation and northern blot analysis

For extraction of genomic DNA, mycelia were homogenized and DNA was isolated according to Sambrook et al. (1989). RNA was isolated using TRI Reagent (Sigma) and peqGOLD Phase Trap (peqlab) reaction tubes. For Northern blot analysis, 10 μg of total RNA were analyzed as described in Oberegger et al. (2001). Hybridization probes were amplified by PCR using the primers listed in Table S2.

### Deletion of *amcA* (AFUA_8G02760) and reconstitution of the Δ*amcA* strain

For generating the Δ*amcA* mutant strain, the bipartite marker technique was used (Nielsen et al., 2006). *A. fumigatus* AfS77 (ATCC46645 Δ*Ku70*) was co-transformed with two DNA fragments, each containing overlapping but incomplete fragments of the pyrithiamine resistance-conferring *ptrA* gene, fused to 1.4 kb *amcA* 5′- and 1.6 kb *amcA* 3′-flanking sequences, respectively. The *amcA* 5′-flanking region (1354 bp) was PCR-amplified from genomic DNA using primers oamcA-1 and oamcA-2. For amplification of the *amcA* 3′-flanking region (1630 bp) primers oamcA-3 and oamcA-4 were employed. Subsequent to gel-purification, these fragments were digested with *Spe*I (5′-flanking region) and *Xho*I (3′-flanking region), respectively. The *ptrA* selection marker was released from plasmid pSK275 (gift from Sven Krappmann, Goettingen, Germany) by digestion with *Spe*I and *Xho*I, and ligated with the 5′- and 3′-flanking region. The transformation construct A (2640 bp, fusion of the *amcA* 5′-flanking region and the *ptrA* split marker) was amplified from the ligation product using primers oamcA-5 and optrA-2.1. For amplification of the transformation construct B (2667 bp, fusion of the *amcA* 3′-flanking region and the supplementary *ptrA* split marker) primers oamcA-6 and optrA-1 were employed. For transformation of *A. fumigatus* AfS77 both constructs A and B were simultaneously used. This strategy deleted the sequence -33 to 1049 bp relative to the translation start site in *amcA*.

For reconstitution of the Δ*amcA* strain with a functional *amcA* copy, a 3987 bp PCR fragment was generated with primers oamcA-1 and oamcA-6 and was subsequently subcloned into a *Stu*I digested pAN7.1 plasmid (containing the hygromycin B resistance-conferring *hph* gene), resulting in plasmid pAN7.1_amcAreverse. The resulting 10.7 kb plasmid was linearized with *Sfi*I and used to transform *A. fumigatus* Δ*amcA*.

Transformation of *A. fumigatus* was carried out as described in Tilburn et al. (1983). For selection of transformants, 0.2 mg ml^−1^ hygromycin B (Calbiochem) was used. To obtain homokaryotic transformants, colonies from single homokaryotic spores were picked. Screening of transformants and confirmation of single genomic integration was performed by PCR and confirmed by Southern blot analysis. Hybridization probes were amplified by PCR using the primers listed in Table S2. Primers used for generating both Δ*amcA* and Δ*amcA*^*c*^ are listed in Table S3.

### Eflornithine inhibition assay

Effects of eflornithine (Sigma-Aldrich) on fungal growth were analyzed by agar diffusion assays, which were carried out as described previously by Beckmann et al. (2013), with slight modifications. As the mutant strain analyzed in this study was not arginine auxotrophic, arginine supplementation was omitted.

### *Galleria Mellonella* virulence assays

Comparison of the virulence potential of *A. fumigatus* wt and Δ*amcA* mutant strain in the *G. mellonella* model was carried out according to Fallon et al. (2011), and as described previously by Beckmann et al. (2013).

### Statistical analyses

For statistical analyses, an unpaired *t*-test was used, comparing mutant data with data from wt and the complemented strain. Significance levels are as follows: ^*^indicates a *p*-value lower than 0.05 while ^**^represent a *p*-value below 0.01.

## Results/discussion

### Inactivation of amcA reduces radial growth on glutamine and particularly ornithine but not arginine as nitrogen source

**Figure 1:**
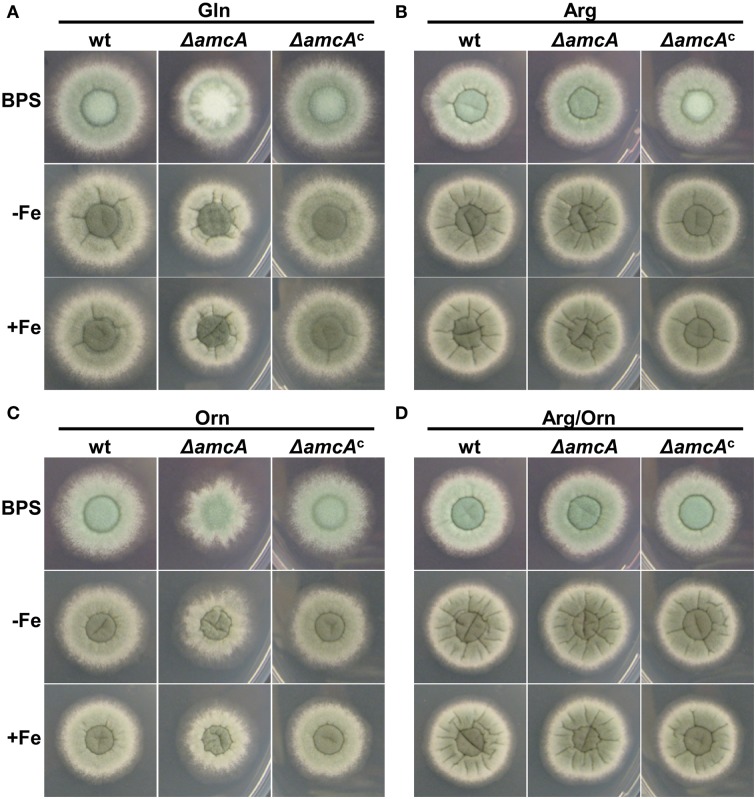
**AmcA-deficiency reduces radial growth on glutamine (Gln) or ornithine (Orn) but not arginine (Arg) or Arg/Orn**. Fungal growth on solid medium was analyzed after 48 h on plates containing 20 mM Gln **(A)**, 20 mM Arg **(B)**, 20 mM Orn **(C)** or 10 mM Arg/10 mM Orn **(D)**. BPS plates contained 0.2 mM BPS to increase iron starvation. For -Fe plates, iron was omitted. Iron-replete plates (+Fe) contained 30 μM FeSO_4_.

Previously it was shown, that iron deficiency causes an active upregulation of ornithine biosynthesis as well as the cellular ornithine content (Schrettl et al., 2010). This common precursor for all siderophores of *A. fumigatus* is produced either via the conversion of arginine within the cytosol, or in the mitochondria from glutamate (Davis, 1986). The study of arginine auxotrophic mutants impaired (Δ*argEF*) or not impaired (Δ*argB*) in mitochondrial ornithine production indicated a dominant role of the latter in SB of this opportunistic fungal pathogen (Beckmann et al., 2013). To gain more insight into ornithine supply for SB, we compared a mutant lacking the putative ornithine transporter AmcA (Δ*amcA*) with its respective wild type (wt) strain on a variety of different nitrogen sources. Δ*amcA* was generated during this study in the *A. fumigatus* AfS77 strain, a Δ*akuA*-derivative of *A. fumigatus* ATCC 46645, lacking non-homologous recombination (Krappmann et al., 2006). The reconstituted Δ*amcA* mutant, termed Δ*amcA*^*c*^, which was generated by re-integration of a wt gene copy, showed a wt-like phenotype in all experiments confirming that the mutant effects are caused specifically by *amcA* deletion (Figure 1). Plate assays were performed on solid minimal medium, containing glutamine, arginine, ornithine or arginine/ornithine as nitrogen sources, respectively, combined with different iron availability. Growth differences between Δ*amcA* and the wt were compared after spot inoculation with 10^4^ conidia. Figure 1A shows a slight reduction in radial growth of Δ*amcA* with glutamine as the sole nitrogen source independent of the iron supply as well as reduced conidiation under iron starvation conditions (BPS, bathophenanthroline disulfonate). BPS is a ferrous iron-specific chelator, which blocks RIA, thereby rendering siderophore mediated iron uptake the only high-affinity uptake system of *A. fumigatus* (Schrettl et al., 2004). Ornithine as nitrogen source led to strongly reduced radial growth independent of the iron supply (Figure 1C). With arginine, be it either in combination with ornithine or as sole nitrogen source, Δ*amcA* displayed wt-like radial growth and conidiation (Figures 1B,D).

These data indicate that AmcA is important for adaptation to iron starvation (sporulation defect on BPS) on glutamine but not ornithine or arginine as nitrogen source. Moreover, AmcA is required for optimal utilization of glutamine and particularly ornithine as nitrogen source. Taking into account the homology of AmcA with predicted mitochondrial ornithine transporters (*S. cerevisiae* Arg11, *N. crassa* Arg13, *H. sapiens* ORNT1), the defects observed are most likely caused by defective ornithine trafficking between the mitochondria and the cytosol. The sporulation defect on glutamine during iron starvation (BPS) (Figure 1A) might be due to decreased ornithine supply for synthesis of the intracellular siderophore FC, which was previously shown to be crucial for intracellular iron distribution and in consequence for conidiation (Wallner et al., 2009). The latter would be consistent with a role in mitochondrial ornithine export for SB.

### In liquid cultures, AmcA-deficiency decreases growth on ornithine and production of siderophores with glutamine as nitrogen source

**Figure 2:**
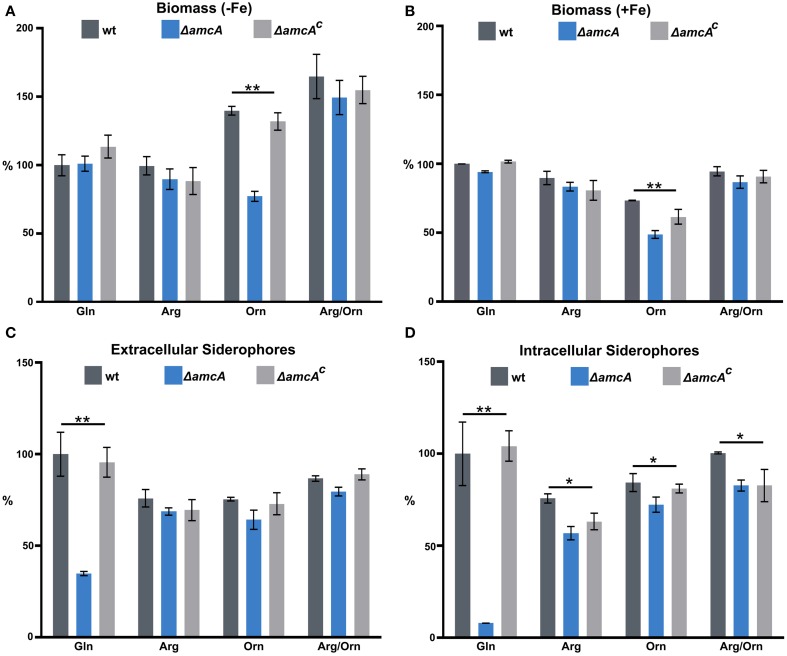
**In liquid cultures, AmcA-deficiency decreases growth on ornithine and production of siderophores on glutamine as nitrogen source**. Fungal growth **(A,B)** and siderophore production **(C,D)** was quantified in liquid medium, containing 20 mM Gln, 20 mM Arg, 20 mM Orn or 10 mM Arg/10 mM Orn after 24 h during iron starvation (−Fe) and iron sufficiency (+Fe). Data represent the mean of three biological replicates ± standard deviation normalized to the biomass and to the wt on Gln. The wt biomass amounted to 0.691 g under +Fe and 0.188 g (27%) under −Fe. The differences in biomass production underline the difference in iron supply. (^*^ ≙ *P* < 0.05, ^**^ ≙ *P* < 0.01).

To further characterize the role of AmcA, biomass and siderophore production of wt, Δ*amcA* and Δ*amcA*^*c*^ were analyzed after growth in liquid cultures with glutamine, arginine, ornithine or arginine/ornithine as nitrogen sources during iron limitation and iron sufficiency (Figure 2). Biomass production displayed a clear correlation with the iron supply as the wt biomass production decreased to 27% during iron starvation conditions compared to iron sufficiency (see legend of Figure 2). In all these assays Δ*amcA*^*c*^ was wt-like. In contrast to the phenotype on solid medium, Δ*amcA* did not display a growth defect on glutamine, neither during iron starvation nor iron sufficiency (Figures 2A,B). Similar to growth on solid media, Δ*amcA* displayed wt-like growth on arginine and arginine/ornithine (Figures 2A,B). In agreement with the growth on solid media, Δ*amcA* showed markedly reduced growth with ornithine: 55% and 67% of the wt during iron starvation and sufficiency, respectively (Figures 2A,B), confirming that AmcA is required for optimal utilization of ornithine as nitrogen source, possibly for mitochondrial ornithine import. Interestingly, the wt showed increased growth on ornithine and arginine/ornithine during iron starvation compared to glutamine or arginine (Figure 2A). Inspection of the ornithine source proved that it was iron-contaminated, indicating that the increased biomass formation on this nitrogen source was a direct consequence of increased iron supply. Nevertheless, ornithine cultures still reflected iron starvation conditions as evident from the high siderophore production (Figures 2C,D) and Northern analysis of iron starvation-induced genes (**Figure 4**). In contrast, during iron sufficiency ornithine led to lower biomass production compared to the other nitrogen sources. These data demonstrate that iron was indeed growth limiting during the iron starvation conditions used, while nitrogen was limiting during the iron sufficient conditions used. Noteworthy, during iron starvation with glutamine as nitrogen source, AmcA-deficiency decreased production of extra- and intracellular siderophores to 35% and 8%, respectively, compared to wt (Figures 2C,D). On arginine, ornithine or arginine/ornithine, AmcA-deficiency decreased the production of extra- and intracellular siderophores only slightly to 80–95% of the wt. These data strongly indicate that AmcA is involved in ornithine supply for SB mainly on glutamine and to a lesser degree on the other nitrogen sources tested.

Consequently, the main function of AmcA on glutamine during iron starvation is most likely the export of mitochondrial ornithine into the cytosol. In line, the defective siderophore production of Δ*amcA* was largely rescued by supplementation with ornithine or arginine, which is hydrolyzed to ornithine by the arginase in the cytosol. Obviously, the reduced siderophore production of Δ*amcA* on glutamine is sufficient to support wt-like growth during iron starvation (Figure 2A). Either the residual siderophore production is sufficient for iron supply or the defect is compensated by reductive iron assimilation, the alternative high affinity iron uptake system (Schrettl et al., 2004). The reduced production of the intracellular siderophore FC is the most likely reason for the observed conidiation defect on BPS-solid medium with glutamine as nitrogen source (see above). Noteworthy, AmcA-deficiency led to decreased sporulation in the presence of BPS as well as decreased extra- and intracellular siderophore production with ammonium tartrate or sodium nitrate as nitrogen sources (Figure S1), as seen with glutamine.

### Ornithine as nitrogen source decreases TAFC and increases FsC production

**Figure 3:**
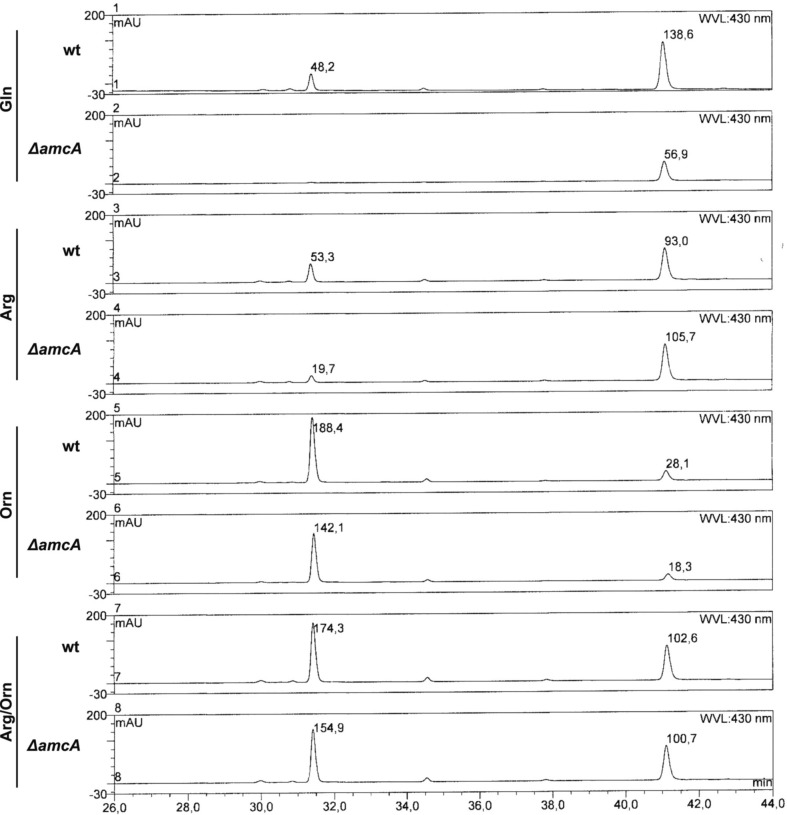
**Reversed-phase HPLC analysis of wt and ΔamcA grown with different nitrogen sources (20 mM Gln, 20 mM Arg, 20 mM Orn, 10 mM Arg/10 mM Orn)**. Ornithine as nitrogen source leads to a shift in siderophore composition from TAFC toward FsC. The peaks at 31.5 and 41 min represent TAFC and FsC, respectively. Absorbance of selected peaks is indicated.

Remarkably, the reversed-phase HPLC-guided analysis of siderophores revealed an influence of the nitrogen source on the composition of extracellular siderophores of *A. fumigatus* wt (Figure 3). As shown previously (Schrettl et al., 2007), *A. fumigatus* produced two extracellular siderophores, TAFC and FsC. On glutamine and arginine, TAFC exceeded FsC by about 3- and 2-fold, respectively. In contrast, on ornithine and arginine/ornithine, FsC surpassed TAFC levels about 7- and 2-fold. Iron supplementation to a final concentration of 1 μM of glutamine cultures did not influence the TafC/FsC ratio, ruling out the possibility that the slight iron contamination of ornithine (see above) is responsible for the decreased of TAFC/FsC ratio in ornithine cultures. Taken together, these data reveal a negative influence of ornithine on TAFC production.

### Iron starvation upregulates *amcA* expression independently of the nitrogen source; ornithine downregulates expression of the SB gene *sidG*

**Figure 4:**
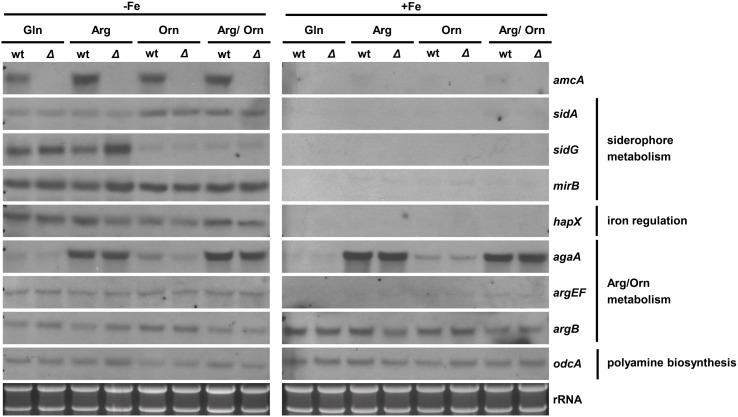
**Iron starvation upregulates *amcA* expression independent of the nitrogen source and AmcA-deficiency downregulates expression of the siderophore biosynthetic gene *sidG***. Northern analysis was performed with RNA from 18 h liquid cultures representing iron starvation (−Fe) as well as iron replete conditions (+Fe) with different nitrogen sources (20 mM Gln, 20 mM Arg, 20 mM Orn, 10 mM Arg/10 mM Orn).

To analyze possible effects of AmcA-deficiency on expression of genes involved in siderophore and arginine/ornithine metabolism, Northern blot analysis of selected genes was performed from iron-starved as well as iron replete cultures (Figure 4). Previously, expression of *amcA* (AFUA_8G02760) was found to be upregulated by iron starvation with glutamine as nitrogen source (Schrettl et al., 2010). Here we found that this transcription pattern also holds true for arginine, ornithine and arginine/ornithine as nitrogen sources (Figure 4). As the latter nitrogen sources render the necessity for mitochondrial ornithine export redundant, these data indicate additional functions of AmcA, such as mitochondrial ornithine import as discussed above. The lack of *amcA* transcript detection in Δ*amcA* confirmed the deletion of the gene. The following genes involved in adaptation to iron starvation (Schrettl et al., 2004, 2008, 2010; Raymond-Bouchard et al., 2012), *sidA* (AFUA_2G07680, an ornithine monooxygenase catalyzing the first committed enzymatic step in the SB pathway), *mirB* (AFUA_3G03640, a siderophore transporter), *hapX* (AFUA_5G03920, the iron regulatory bZip transcription factor) and *sidG* (AFUA_3G03650, the *N*^*2*^-transacetylase) were upregulated during iron starvation and unaffected by AmcA-deficiency (Figure 4). The fact that AmcA-deficiency did not affect transcript levels of any of the analyzed SB genes indicates that the decreased SB of Δ*amcA* with glutamine as nitrogen source has other reasons. Both ornithine and arginine/ornithine as nitrogen source did not significantly affect expression of *mirB* or *hapX*, mildly upregulated *sidA*, but downregulated *sidG*. As *sidG* encodes the enzyme that is responsible for conversion of FsC into TAFC, these data explain the increase of FsC and decrease of TAFC in the presence of ornithine or arginine/ornithine (Figure 3). The reason for the negative effect of ornithine on *sidG* expression and consequently TAFC production remains elusive. As expected, the presence of arginine upregulated the arginase-encoding gene *agaA* (AFUA_3G11430) during both iron starvation and sufficiency, largely unaffected by AmcA-deficiency (Figure 4). These data are in line with arginine catabolism and cytosolic ornithine production under these growth conditions. Expression of the ornithine-biosynthetic gene *argEF* (AFUA_6G02910, acetylglutamate kinase/N-acetyl-γ-glutamyl-phosphate reductase) was upregulated during iron starvation compared to iron sufficiency, as previously reported (Schrettl et al., 2007), and unaffected by AmcA-deficiency. Expression of the arginine-biosynthetic gene *argB* (AFUA_4G07190, ornithine carbamoyltransferase) and the ornithine decarboxylase encoding *odcA* (AFUA_4G08010), which is responsible for the first step in the conversion of ornithine to polyamines, were largely unaffected by both AmcA-deficiency and nitrogen source (Figure 4) (Oberegger et al., 2001; Jin et al., 2002; Beckmann et al., 2013).

Taken together with the data obtained by Northern blot analysis, which indicated wt-like expression of selected SB genes in Δ*amcA* (Figure 4), the decrease of SB of Δ*amcA* with glutamine as nitrogen source is most likely caused by the decrease in the cellular ornithine pool. Moreover, AmcA-deficiency does not appear to affect arginine/ornithine biosynthesis at the transcriptional level.

### AmcA-deficiency decreases the cellular pool of arginine and particularly ornithine during iron starvation

Effects of AmcA-deficiency on amino acid composition were analyzed by comparison of the free amino acid pools of wt and Δ*amcA* in iron-starved and iron replete liquid cultures with 20 mM glutamine supplementation (Table 1). As previously shown for another *A. fumigatus* strain (Schrettl et al., 2010), iron starvation dramatically altered the composition of the free amino acid pool, including a 16-fold increase in both ornithine and arginine (Table 1). In comparison to the wt, during iron starvation AmcA-deficiency decreased the levels of arginine and ornithine by 48% and 84%, respectively, with the effect on the cellular ornithine content being the most severe of all amino acids.

**Table 1:** **Analysis of the amino acid composition of the wt and mutant strain under iron-replete and iron depleted conditions with glutamine as nitrogen source after 24 h of growth in % of total free amino acids**.

**aa**	**Wt**			**Δ*amcA***			**Δ*amcA*/wt**	
	**+Fe**	**−Fe**	**−/+Fe**	**+Fe**	**−Fe**	**−/+Fe**	**+Fe**	**−Fe**
Ala	15.75 ± 0.27	4.51 ± 0.13	***0.29***	20.87 ± 0.51	6.10 ± 0.22	***0.29***	1.33	1.35
Arg	1.05 ± 0.03	16.67 ± 0.16	***15.94***	0.86 ± 0.01	8.61 ± 0.22	***9.96***	0.83	**0.52**
Asn	0.72 ± 0.03	3.45 ± 0.11	***4.78***	0.80 ± 0.02	3.28 ± 0.10	***4.11***	1.11	0.95
Asp	16.80 ± 0.14	4.47 ± 0.13	***0.27***	7.49 ± 0.09	4.58 ± 0.19	**0.61**	**0.45**	1.03
Gln	9.17 ± 0.22	36.94 ± 0.33	***4.03***	5.11 ± 0.38	48.17 ± 0.45	***9.42***	**0.56**	1.30
Glu	44.58 ± 0.61	15.95 ± 0.42	**0.36**	53.60 ± 0.28	18.41 ± 0.73	**0.34**	1.20	1.15
Gly	0.98 ± 0.07	1.23 ± 0.07	1.26	1.00 ± 0.05	0.82 ± 0.03	0.82	1.02	**0.66**
His	0.57 ± 0.01	2.73 ± 0.07	***4.80***	0.60 ± 0.02	2.80 ± 0.00	***4.68***	1.05	1.03
Ile	0.51 ± 0.06	0.23 ± 0.02	**0.45**	0.43 ± 0.00	0.22 ± 0.01	**0.50**	0.83	0.93
Leu	0.59 ± 0.01	0.32 ± 0.01	**0.54**	0.42 ± 0.01	0.20 ± 0.00	**0.47**	0.72	**0.63**
Lys	1.40 ± 0.07	4.28 ± 0.04	***3.06***	1.83 ± 0.04	2.13 ± 0.06	1.17	1.31	**0.50**
Met	0.09 ± 0.02	0.11 ± 0.01	1.24	0.07 ± 0.03	0.06 ± 0.02	0.80	0.86	**0.56**
Orn	0.30 ± 0.01	5.11 ± 0.11	***16.78***	0.45 ± 0.04	0.83 ± 0.00	**1.86**	1.47	***0.16***
Phe	0.24 ± 0.02	0.13 ± 0.02	**0.55**	0.17 ± 0.01	0.10 ± 0.01	**0.57**	0.72	0.74
Ser	2.49 ± 0.05	1.52 ± 0.03	**0.61**	2.16 ± 0.04	1.37 ± 0.05	**0.63**	0.87	0.90
Thr	3.31 ± 0.03	1.35 ± 0.05	**0.41**	2.08 ± 0.00	1.52 ± 0.07	0.73	**0.63**	1.12
Trp	0.00 ± 0.00	0.00 ± 0.00	0.00	0.00 ± 0.00	0.00 ± 0.00	0.00	0.00	0.00
Tyr	0.29 ± 0.01	0.39 ± 0.01	1.37	0.29 ± 0.03	0.31 ± 0.00	1.07	1.01	0.79
Val	1.15 ± 0.03	0.60 ± 0.03	0.52	1.76 ± 0.01	0.49 ± 0.01	***0.28***	**1.53**	0.81

Individual amino acid pools are given in % of the total free amino acids. Amino acid pools up-regulated >1.5- and >3-fold are in ***bold*** and ***bold/italics*** respectively. Down-regulation of >1.5- and >3-fold is marked in ***bold*** and ***bold/italics***. Given values represent the mean of two biological replicates ± standard deviation.

A significant decrease of arginine and particularly ornithine pools was previously also found for the *S. cerevisiae* mutant lacking the AmcA ortholog Arg11 (Crabeel et al., 1996). These data stress the role of AmcA as a mitochondrial ornithine transporter. The possible reasons for the decreased arginine pool include increased conversion of arginine to ornithine via arginase, although not visible at the transcript level of arginase-encoding *agaA* in Northern blot analysis (Figure 4). Most likely, the decreased cellular ornithine and arginine levels are caused by feed-back inhibition of mitochondrial arginine/ornithine biosynthetic enzymes due to the increased mitochondrial ornithine content resulting from blocked ornithine export. The change in content of the other amino acids underlines the interconnection of metabolic pathways.

### AmcA-deficiency mildly decreases polyamine levels

Polyamines (putrescine, spermine and spermidine) are organic cations required for normal growth as well as cell proliferation and differentiation of all organisms (McCann et al., 1987; Hu and Pegg, 1997; Pegg, 2009). After having observed decreased ornithine, in addition to decreased siderophore levels under glutamine supplementation, we considered it worthwhile to have a closer look at polyamine levels in the Δ*amcA* mutant, as ornithine is the precursor for biosynthesis of not only siderophores, but also polyamines. In the first and rate-limiting step, ornithine is converted via the ornithine decarboxylase to putrescine, which is then further converted to spermidine, the major polyamine in *Aspergilli*, and later to spermine (Jin et al., 2002). The analysis confirmed spermidine as the major polyamine of *A. fumigatus* under iron-replete as well as iron depleted conditions (Table 2). During iron starvation, the total polyamine content of *A. fumigatus* wt decreased to 47% compared to iron-replete conditions (Table 2). AmcA-deficiency mildly decreased the total polyamine content compared to wt, to 83% and 87% during iron sufficiency and iron starvation, respectively (Table 2).

**Table 2:** **Analysis of polyamine production in wt and Δ*amcA* under iron-replete and iron depleted conditions after 24 h cultivation with glutamine as nitrogen source**.

**Polyamines**	**wt**		**Δ*amcA***	
	**+Fe**	**−Fe**	**+Fe**	**−Fe**
Putrescine	20.94 ± 18.71	9.39 ± 10.75	7.31 ± 2.09	5.07 ± 0.85
Spermidine	294.39 ± 32.40	105.45 ± 16.40	231.42 ± 3.59	99.04 ± 21.73
Spermine	64.15 ± 1.46	63.39 ± 1.65	77.11 ± 16.42	51.26 ± 21.70
Total	**379.48**	**178.23**	**315.84**	**155.37**

The values are given in nmol/ml and represent the mean of two biological replicates ± standard deviation.

These data indicate cellular prioritization of ornithine flux into polyamine rather than SB as the experiments described above revealed a significant decrease of the cellular ornithine pool and siderophore production in Δ*amcA* (Table 1 and Figure 2). These observations underline the importance of fine-tuned cellular polyamine levels within an organism. Of course iron supply is as essential as polyamine biosynthesis. However, *A. fumigatus* harbors an ornithine-independent, high-affinity iron uptake system, namely RIA (Schrettl et al., 2004), while there is no alternative for polyamine biosynthesis. This might explain the prioritization, which was also indicated in the study investigating the link between arginine/ornithine metabolism and SB using arginine auxotrophic mutants (Beckmann et al., 2013).

### AmcA-deficiency leads to increased sensitivity against the ornithine decarboxylase (OdcA) inhibitor eflornithine

**Figure 5:**
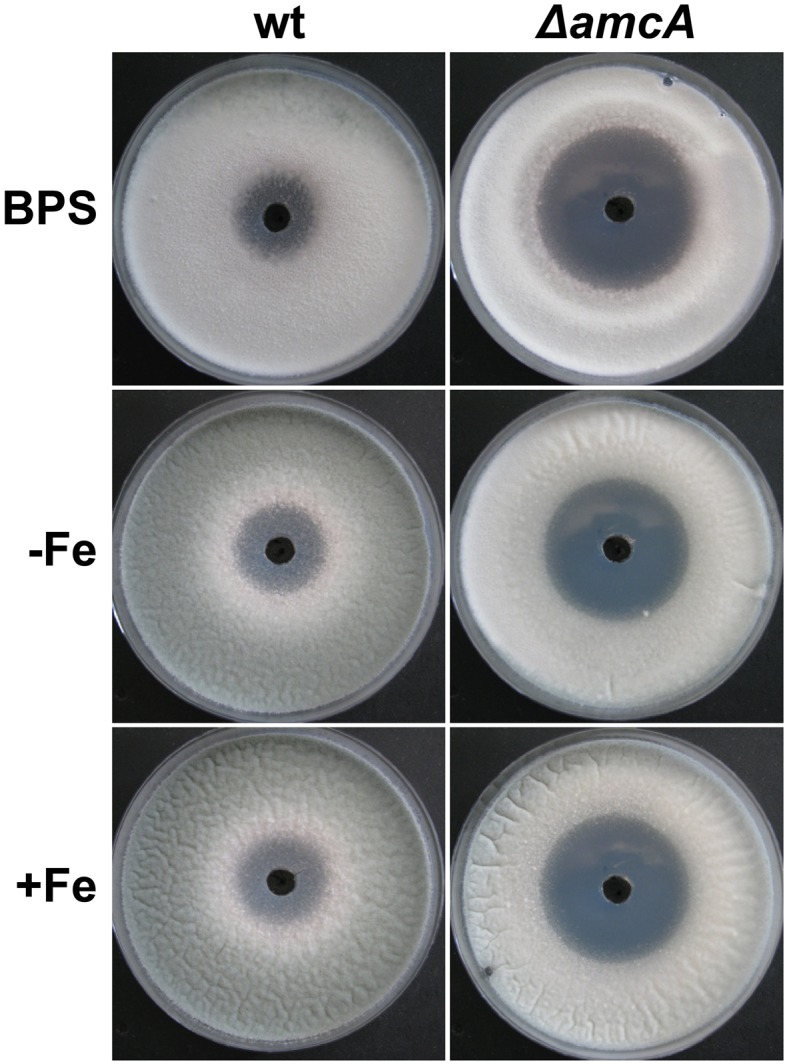
**Deficiency in AmcA increased susceptibility to eflornithine under different availability for iron (BPS, −Fe, +Fe) with glutamine as nitrogen source**. 100 μl of a 0.3 M eflornithine solution were added to a hole pricked in the middle of the plate. Growth inhibition on solid medium was scored after 48 h.

To further analyze the link between AmcA-deficiency, the ornithine pool and polyamine homeostasis, we analyzed the effect of AmcA-deficiency on susceptibility to eflornithine (α-difluormethylornithine) using plate-diffusion assays (Figure 5). Eflornithine is the most effective inhibitor of the ornithine decarboxylase and is used in treatment against African trypanosomiasis and hirsutism (Pegg, 1986; Ramot et al., 2010; Kennedy, 2013). Confirming previous data (Beckmann et al., 2013), *A. fumigatus* is susceptible to eflornithine under iron sufficiency and iron starvation (Figure 5). However, AmcA-deficiency significantly increased susceptibility to eflornithine (inhibition zone of 40 mm of Δ*amcA* compared to 23 mm of wt) (Figure 5).

These data indicate that inhibition of the ornithine decarboxylase is more detrimental to Δ*amcA* than the wt, which is most likely a consequence of the decreased ornithine content (Table 1).

### Deficiency in AmcA does not impede virulence of *A. fumigatus* in an insect host model

**Figure 6:**
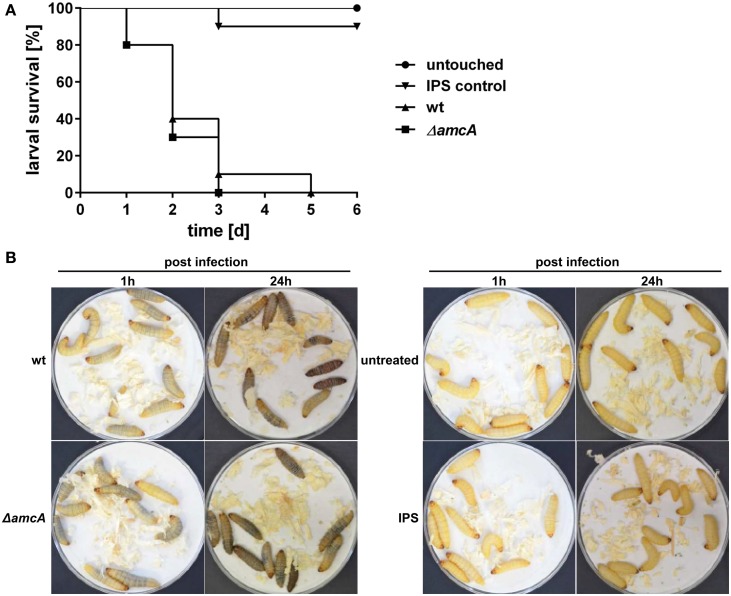
**Deficiency in AmcA does not attenuate virulence of *A. fumigatus* in the *Galleria mellonella* infection model**. **(A)** No significant difference in virulence between Δ*amcA* and wt could be detected (*P* = 0.5253). Insect physiological saline (IPS) was used as an injection control. 90% of all larvae in this group remained viable for the entire experiment. **(B)** Exemplary infection assay with *Galleria mellonella larvae*. Δ*amcA* and wt (AfS77) exhibit same rate of melanisation 1 h and 24 h post infection, indicating progression of infection, and resulting in 100% mortality at day 3 and 5, respectively **(A)**.

In order to assess the role of AmcA in the virulence of *A. fumigatus*, we compared the Δ*amcA* mutant with the wt strain in the *Galleria mellonella* infection model (Fallon et al., 2011). AmcA does not seem to play a major role in the pathogenicity of *A. fumigatus*, as AmcA-deficiency resulted in no significant differences in survival rates (*P* = 0.5253) over a period of 6 days (Figures 6A,B).

Previously, defective SB was shown to result in attenuated virulence in different models, including pulmonary and keratitis mouse models as well as *G. mellonella* (Schrettl et al., 2004, 2007; Slater et al., 2011; Yasmin et al., 2012; Beckmann et al., 2013; Leal et al., 2013). The dramatic decrease in siderophore production of the Δ*amcA* mutant with glutamine as nitrogen source might have led to speculate about an attenuated virulence of this strain. However, previous studies have demonstrated wt-like virulence of an *A. fumigatus argB* mutant in *G. mellonella* (Beckmann et al., 2013), which indicates arginine availability in the host niche of this virulence model. The latter might explain wt-like virulence of Δ*amcA* as arginine restored SB in Δ*amcA* (Figure 2). Alternatively, or combined with the arginine availability, the residual siderophore production of Δ*amcA* could be the reason for unaltered virulence of *A. fumigatus ΔamcA*.

## Conclusion

**Figure 7:**
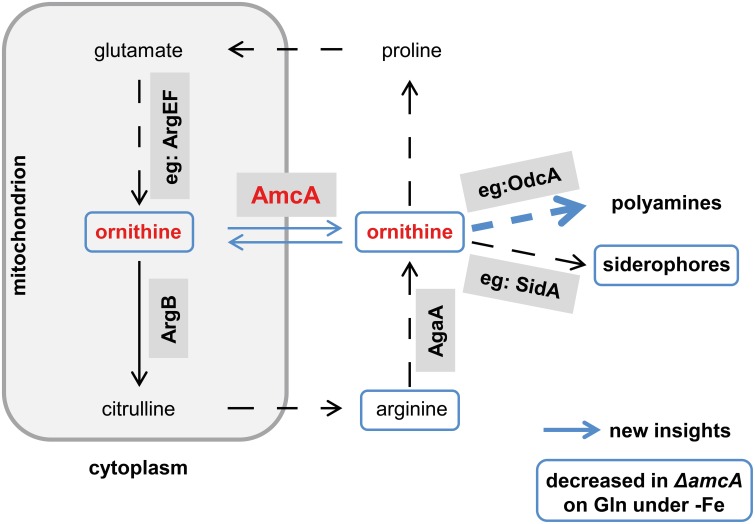
**Postulated working model for AmcA function**. This schematic view of the link between AmcA, ornithine and siderophore production was adopted from Beckmann et al. (2013). Novel findings of this study are marked in blue. Whether the decreased cellular ornithine level shown in Table 1 is caused mitochondrially or cytosolically remains to be elucidated. Prioritization of polyamine synthesis over siderophore production is indicated by the bulky blue arrow.

This study identified a novel cellular component supporting SB of *A. fumigatus*. Despite the lack of direct prove, several lines of evidence indicate a function of AmcA in the supply of the precursor ornithine via mitochondrial ornithine export: (i) the AmcA protein is a member of the mitochondrial carrier protein family with homologs involved in ornithine exchange between the mitochondrial matrix and the cytosol, (ii) AmcA-deficiency in *A. fumigatus* resulted in a dramatically decreased cellular ornithine content and SB during iron starvation with glutamine as nitrogen source but not ornithine or arginine, which stresses cytosolic ornithine production, (iii) AmcA-deficiency led to mildly decreased cellular polyamine content and significantly increased susceptibility to the polyamine biosynthesis inhibitor eflornithine. The novel findings and the postulated function of AmcA are summarized in Figure 7.

In line with its role in precursor supply for siderophore biosynthesis, *amcA* was transcriptionally upregulated during iron starvation. The fact that this upregulation was independent of the nitrogen source (e.g., with arginine and ornithine) indicates additional roles of AmcA such as mitochondrial ornithine import. In line, AmcA-deficiency impaired growth on ornithine as nitrogen source.

In the *G. mellonella* model, AmcA-deficiency did not affect virulence of *A. fumigatus*, most likely due to the residual siderophore production and/or arginine availability in this host niche.

### Conflict of interest statement

The authors declare that the research was conducted in the absence of any commercial or financial relationships that could be construed as a potential conflict of interest.

## Supplementary material

Figure S1 **(A)** AmcA-deficiency decreases radial growth independent of the iron supply and sporulation in the presence of BPS on 20 mM glutamine (Gln), 20 mM ammonium tartrate (ammonium), and 70 mM sodium nitrate (nitrate) as nitrogen sources. Fungal growth on solid medium was analyzed after 48 h on plates. BPS plates contained 0.2 mM BPS to increase iron starvation. For −Fe plates, iron was omitted. Iron-replete plates (+Fe) contained 30 μM FeSO_4_. **(B,C)** Fungal growth was quantified in liquid culture, containing 20 mM glutamine (Gln), 20 mM ammonium tartrate (ammonium) or 70 mM sodium nitrate (nitrate) after 24 h during iron starvation (−Fe) and iron sufficiency (+Fe), respectively. **(D,E)** AmcA-deficiency decreases production of extra- and intra-cellular siderophores on glutamine, ammonium tartrate and sodium nitrate as nitrogen sources. Data represent the mean of three biological replicates ± standard deviation normalized to the biomass and to the wt on Gln. (^*^ ≙ *P* < 0.05, ^**^ ≙ *P* < 0.01).
