# Supplementary material for: AmcA—a putative mitochondrial ornithine transporter supporting fungal siderophore biosynthesis
Source: Front Microbiol. 2015 Apr 7;6:252. doi: 10.3389/fmicb.2015.00252 (PMC4387927; doi:10.3389/fmicb.2015.00252)
Supplement: Supplementary file 2 [file Table1.DOCX]

**Table S1:** Fungal strains used in this study.

| **Strain** | **Description** | **Reference** |
| --- | --- | --- |
| **AfS77**  ***ΔamcA***  ***ΔamcA^c^*** | ATCC46645 *ΔKu70*  *amcA*(AFUA8_G02760)*::ptrA; AfS77*  *amcA::ptrA; AfS77; amcA* | Sven Krappmann  This study  This study |
