# Supplementary material for: AmcA—a putative mitochondrial ornithine transporter supporting fungal siderophore biosynthesis
Source: Front Microbiol. 2015 Apr 7;6:252. doi: 10.3389/fmicb.2015.00252 (PMC4387927; doi:10.3389/fmicb.2015.00252)
Supplement: Supplementary file 3 [file Table2.DOCX]

**Table S2:** Primers used for amplification of hybridization probes.

| **Primer** | **gene** | **Sequence 5’-3’** |
| --- | --- | --- |
| **oAfAmcA-f** | **mitochondrial ornithine carrier AmcA** | TCA ATG GAG CTG CCT GTC |
| **oAfAmcA-r** |  | CAA TTC CGT AGC CCT TCG |
| **osidA1** | **L-ornithine monooxygenase SidA** | AAC TAC CTC CAC CAG AAG |
| **osidA2** |  | GAA CGG CAA TGT TGT AAG |
| **oAfAT1me** | **N^2^-transacetylase** | ACA ATC AAG GCT CAG CCC |
| **oAfAT2me** |  | ACT TCG AGT CAT GCT GGG |
| **oAfmirB1me** | **siderophore transporter MirB** | AAG CCG AGA AAA AGG GGG |
| **oAfmirB2me** |  | AAC CCA GAT GAA GCC CAG |
| **oAfhapX-Seq.1** | **bZip transcription factor HapX** | TAC CAT TCT CCT CCA CCC |
| **oAfhapXseq.r** |  | CGA CGA TGT ATT GTT ATT GG |
| **oAfArg-f** | **arginase** | ACC GCA CCA AAG AGC AAC |
| **oAfArg-r** |  | CGA CGG AGG AAG GAA ATC |
| **oAfArg5,6-f** | **acetylglutamate kinase** | TTC GTA CTC GCC ATA GCC |
| **oAfArg5,6-r** |  | CTT CTC AAT GCT CAC CCC |
| **oAoArgB1.f** | **ornithine carbamoyltransferase ArgB** | CTT CTC CGC ATA CTA CCG |
| **oAoArgB1.r** |  | CCC AAA CTG TCA GAG CGA |
| **oAfdCarb-f** | **ornithine decarboxylase ODC** | AAA GTA CAG CCA GTC GCC |
| **oAfdCarb-r** |  | AAT CAT GGA GGG GAC GAC |
