# Supplementary material for: AmcA—a putative mitochondrial ornithine transporter supporting fungal siderophore biosynthesis
Source: Front Microbiol. 2015 Apr 7;6:252. doi: 10.3389/fmicb.2015.00252 (PMC4387927; doi:10.3389/fmicb.2015.00252)
Supplement: Supplementary file 4 [file Table3.DOCX]

**Table S3:** Primers used for generation of *ΔamcA* and *ΔamcA^c^.*

| **Primer** | **Sequence 5’-3’** | |  |
| --- | --- | --- | --- |
| **oamcA-1** | CCT GCA TCG CCG CCA ATT |  | |
| **oamcA-2**  **oamcA-3**  **oamcA-4**  **oamcA-5**  **oamcA-6** | CAA ACT AGT GTG CAG GTG ATG GAA GAG  GAT CTC GAG TTG CCC GTG TCT TCC CAT  TGA CAT CGG CTG CTG ATC  CGC TTC TCC CTT CGC TTT  GAG ACC CAA CTC AGT AGC |  | |
